# Reconstructing the Indian Origin and Dispersal of the European Roma: A Maternal Genetic Perspective

**DOI:** 10.1371/journal.pone.0015988

**Published:** 2011-01-10

**Authors:** Isabel Mendizabal, Cristina Valente, Alfredo Gusmão, Cíntia Alves, Verónica Gomes, Ana Goios, Walther Parson, Francesc Calafell, Luis Alvarez, António Amorim, Leonor Gusmão, David Comas, Maria João Prata

**Affiliations:** 1 Institute of Evolutionary Biology (CSIC-UPF), CEXS-UPF-PRBB, Barcelona, Spain; 2 Institute of Molecular Pathology and Immunology, University of Porto (IPATIMUP), Porto, Portugal; 3 Faculty of Sciences, University of Porto, Porto, Portugal; 4 Institute of Legal Medicine, Innsbruck Medical University, Innsbruck, Austria; 5 CIBER de Epidemiología y Salud Pública, (CIBERESP), Barcelona, Spain; Smithsonian Institution National Zoological Park, United States of America

## Abstract

Previous genetic, anthropological and linguistic studies have shown that Roma (Gypsies) constitute a founder population dispersed throughout Europe whose origins might be traced to the Indian subcontinent. Linguistic and anthropological evidence point to Indo-Aryan ethnic groups from North-western India as the ancestral parental population of Roma. Recently, a strong genetic hint supporting this theory came from a study of a private mutation causing primary congenital glaucoma. In the present study, complete mitochondrial control sequences of Iberian Roma and previously published maternal lineages of other European Roma were analyzed in order to establish the genetic affinities among Roma groups, determine the degree of admixture with neighbouring populations, infer the migration routes followed since the first arrival to Europe, and survey the origin of Roma within the Indian subcontinent. Our results show that the maternal lineage composition in the Roma groups follows a pattern of different migration routes, with several founder effects, and low effective population sizes along their dispersal. Our data allowed the confirmation of a North/West migration route shared by Polish, Lithuanian and Iberian Roma. Additionally, eleven Roma founder lineages were identified and degrees of admixture with host populations were estimated. Finally, the comparison with an extensive database of Indian sequences allowed us to identify the Punjab state, in North-western India, as the putative ancestral homeland of the European Roma, in agreement with previous linguistic and anthropological studies.

## Introduction

The dispersion of the Roma (Gypsies) through Europe represents one of the most remarkable people movements in recent historical times. The current estimates of the total Roma population size in Europe range from 4 to 10 million, with the largest numbers concentrated in Central and South-eastern Europe [1], [2]. The Roma constitute a diasporic population without any reliable written records, neither historic nor genealogic. Mainly of nomadic lifestyle and with endogamous social practices, the geographically dispersed Roma populations have been socially marginalized and historically persecuted [3].

Linguistic, anthropological, historical and genetic evidences point out India as the origin of the Roma populations, which may have left the continent approximately between the 5^th^–10^th^ centuries [3]. After leaving India, the Roma migration route passed through Persia, Armenia, Greece and the Slavic-speaking parts of the Balkans [3]. The acknowledgment of the Roma establishment in the Balkan region is uniformly accepted to have taken place during the 11^th^ and 12^th^ centuries, where they remained for two centuries before they started spreading out to all over Europe [2], [3]. The dispersion throughout the continent was a very fast process since by the 15^th^ century Roma had reached the Northern and Westernmost fringes of Europe. Indeed, historical documents testify that by the early 15^th^ century Roma were present in Catalonia and by the end of the century they were spread all over Spain and Portugal. The most important gateway for the entrance of Roma in Iberia is believed to have been the Trans-Pyrenees route. Three more recent migration waves have to be taken into account in the formation of the present-day Roma populations from Western Europe. First, the dispersion that occurred during the end of the 19th century, after the abolition of Roma slavery in the Romanian Old Kingdom [1], [3], [4]; second, out of Yugoslavia, during the 1960s and 1970s; and third, during the last decade, following the political and economic changes in Eastern Europe [5].

Previous genetic studies have confirmed the Indian origin of the Roma and have also described differential admixture with the European neighboring groups [6], [7], [8], [9], [10]. However, these studies lacked accurate representation of Western Roma groups [6] and it was not until recently that genetic studies on Iberian Roma were published [11], [12]. Nonetheless, the specific origin of the Roma within the Indian continent has not been elucidated yet. Linguistic evidences point out to North-western India as the source of the proto-Roma population, specifically to the Indo-Aryan ethnic groups in that area [4]. Multilocus comparison of classical genetic markers [13] showed strong affinities of the Roma with Rajput and Punjabi populations from North-Western India. Additional genetic evidence relating the Roma populations to this geographical area comes from the study of a private mutation causing primary congenital glaucoma in the Roma which has been also described in a family belonging to the Jatt, an ethnic group of Indo-Aryan descent from the Pakistani Punjab province [14]. In previous studies, the selection of Indian/Pakistani populations was influenced by linguistic theories on the Roma origins and/or by the availability of the genetic data from the Indian subcontinent [15]. Therefore, the need for an unbiased coverage of the Indian genetic data is necessary to locate the place of origin of the Roma Diaspora in the subcontinent.

The present study aims to survey the maternal genetic legacy in the Roma in order to achieve a deeper knowledge of their history. We provide additional 214 mitochondrial DNA (mtDNA) complete control region sequences from Roma individuals from the Iberian Peninsula and analyze them in the context of the previously published studies on other Roma populations. The non-recombinant nature and the phylogeographic resolution of the mtDNA permits not only to survey the genetic affinities among different Roma groups and host populations, but to study the migration routes followed by the Roma and the putative origin of the Roma in the Indian subcontinent.

## Materials and Methods

### Ethic statements

Written informed consent was obtained from the participants and analyses were performed anonymously. The project obtained the ethics approval from the Institutional Review Board of the institutions involved in the sampling (Conselho Nacional de Ética para as Ciências da Vida (CNECV) in Portugal, and Comitè Ètic d'Investigació Clínica – Institut Municipal d'Assistència Sanitària (CEIC-IMAS) in Spain).

### Sample collection

A total of 214 unrelated individuals from the Iberian Peninsula were analyzed. 138 individuals were sampled in Portugal from 18 different communities in 11 districts, whereas 76 subjects were collected in Barrio de la Mina neighborhood in Sant Adrià de Besòs, Barcelona, Catalonia, Spain. All the individuals self-declared as “ciganos/gitanos” (Portugal/Spain) and were interrogated about family history in order to avoid close kinship.

### Mitochondrial DNA amplification and sequencing

DNA was extracted from fresh blood by standard phenol-chloroform method. The complete mitochondrial control region (16024–576 bp) was amplified by PCR using the primers L15997 (5′-CACCATTAGCACCCAAAGCT-3′) and H599 (5′-TTGAGGAGGTAAGCTACATA-3′). Both hypervariable segments were sequenced in both directions, for HVR-I (hypervariable region I, positions 16024-16569) the reverse primer was H17 (5′-CCCGTGAGTGGTTAATAGGGT-3′), whereas for HVR-II (positions 1-576) the forward primer was L16555 (5′-CCCACACGTTCCTAAAT-3′). In addition, in the Spanish Roma samples, five Single Nucleotide Polymorphisms (SNPs) in the coding region of mtDNA (H10400, L10873, L12308, L12705 and L11719) were determined by SNaPshot™ ddNTP Primer Extension Kit (Applied Biosystems) as described in Bosch et al. [16]. Two additional SNPs (L7028 and L11251) were genotyped in the sequences classified as HV/H and R/JT respectively.

MtDNA variation was compared to the revised Cambridge Reference Sequence (rCRS) [17] and mtDNA sequences were classified into haplogroups according to Van Oven and Kayser [18]. Samples belonging to haplogroup H or with a dubious ascription to this haplogroup were further genotyped for a set of coding region SNPs [19] in order to refine the classification.

### Statistical Analyses

In order to locate the Iberian Roma in the context of other European Roma and their corresponding host populations, a database of 1,890 hypervariable region I (HVR-I) sequences (positions 16090 to 16365) was built from previously published studies (hereafter referred to as Roma-host database). In addition to the 138 Portuguese and 76 Spanish Roma from this study, the Roma-host database contained other sequences gathered from the literature: 39 Spanish Roma [6], [20], 232 Bulgarian and 18 Lithuanian Roma [6], 69 Polish Roma [9], and 205 Hungarian Roma [7]. To cover the corresponding European host populations, we collected 118 Portuguese individuals (*unpublished data*), 68 Spanish [21], 141 Bulgarian [22], 162 Lithuanian [23], 413 Polish [24], and 211 Hungarian [7]. The Bulgarian Roma populations from Gresham et al. [6] were grouped according to the original paper classification (“Bulgaria 1” stands for Roma groups who settled early in Bulgaria, whereas “Bulgaria 2” and “Bulgaria 3” stand for Roma groups settled in Bulgaria coming originally from Wallachia/Moldavia in the 17th–18th centuries and late 19^th^ century respectively).

Intrapopulation genetic diversity parameters such as number of different sequences (K), sequence diversity values (Ĥ) [25], number of polymorphic sites (S) and nucleotide diversity (π) [25], [26] were calculated for the HVR-I using Arlequin software v3.1 [27]. Additionally the weighted intralineage mean pairwise differences (WIMP) were also computed, which measures mean pairwise differences within each lineage but weighting for its corresponding frequency [28]. Finally the female effective-population sizes were assessed by the computation of the estimators *θ*_π_, *θ*_K_ and *θ*_S_ (*θ = 2N_fe_μ* where *N_fe_* is the female effective-population size and *μ* is the mutation rate). Whereas *θ_S_* is based on the number of segregating sites, *θ_K_* relies on the observed number of different lineages. Since the mutation rate for the HVR-I should be the same in all populations, differences in *θ* values reflect differences in the female effective-population sizes among populations [29].

Pairwise differences between populations were represented in a Non-Metric Multidimensional Scaling plot (NMDS) by using STATISTICA 7 package (http://www.statsoft.com) with default starting configuration.

Population genetic structure was tested through analysis of molecular variance (AMOVA) [30] using Arlequin v3.1 software [27] to shed light on the migration routes that Roma populations may have followed in Europe by comparing country of residence to migration routes.

Taking advantage of the phylogeographic information of the mitochondrial sequences and following the same approach as in Mendizabal et al. [31], admixture between Roma and European host populations was estimated. In addition, several Indian geographic areas were evaluated as possible ancestral homeland of the Roma. Two datasets were compiled for these purposes: the extended database of host European sequences with 5,096 individuals from Iberia, Balkans, Hungary, Poland and Baltic countries (from Additional File 1 in Mendizabal et al. [31]), whereas Indian sequences were collected from Dubut et al. [32] (n = 3,751, excluding Sri Lanka). Each of the datasets was subdivided into subcontinental regions and the probability of origin at each region was calculated as
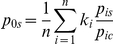
where, *n* is the number of Roma sequences with matches (≥1) in the whole subcontinental dataset of India; k*_i_*, the number of times the sequence *i* is found in the Roma sample; p*_is_*, the frequency of the sequence *i* in the specific region of India; and p*_ic_,* the frequency of the sequence *i* in the whole subcontinental Indian dataset. Standard deviations for each of the estimations were computed as 
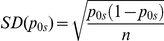



A median-joining network was generated to infer phylogenetic relationships between European Roma and Indian mtDNA lineages (HVR-I, positions 16090–16365) using Network 4.5.0.0 software (http://www.fluxus-engineering.com/). Mutation weights were in accordance with Santos et al. [33], excluding insertions and deletions. The time to the most common ancestor (TMRCA) of M5a1 subhaplogroup was estimated based on the average number of mutations accumulated from an ancestral sequence as a linear function of time and mutation rate. The age estimates were obtained with Network 4.5.0.0 by considering one transition per 18,845 years in the sequence range of 16090–16365 [34].

## Results

### MtDNA diversity and demography estimators in the Roma

A total of 59 distinct sequences were found in the complete mitochondrial control region in the 214 Iberian Roma analyzed (Table S1), out of which only two were shared between Portuguese and Spanish subjects (Hap38 and Hap49). No significant differences were observed between the Spanish Roma from the present study (Barcelona) and those from previously published studies (Andalusia and Madrid) (variance among groups = 0.85%, p-value = 0.173). Therefore, all Spanish Roma samples were considered as a single one.

**Figure 1:**
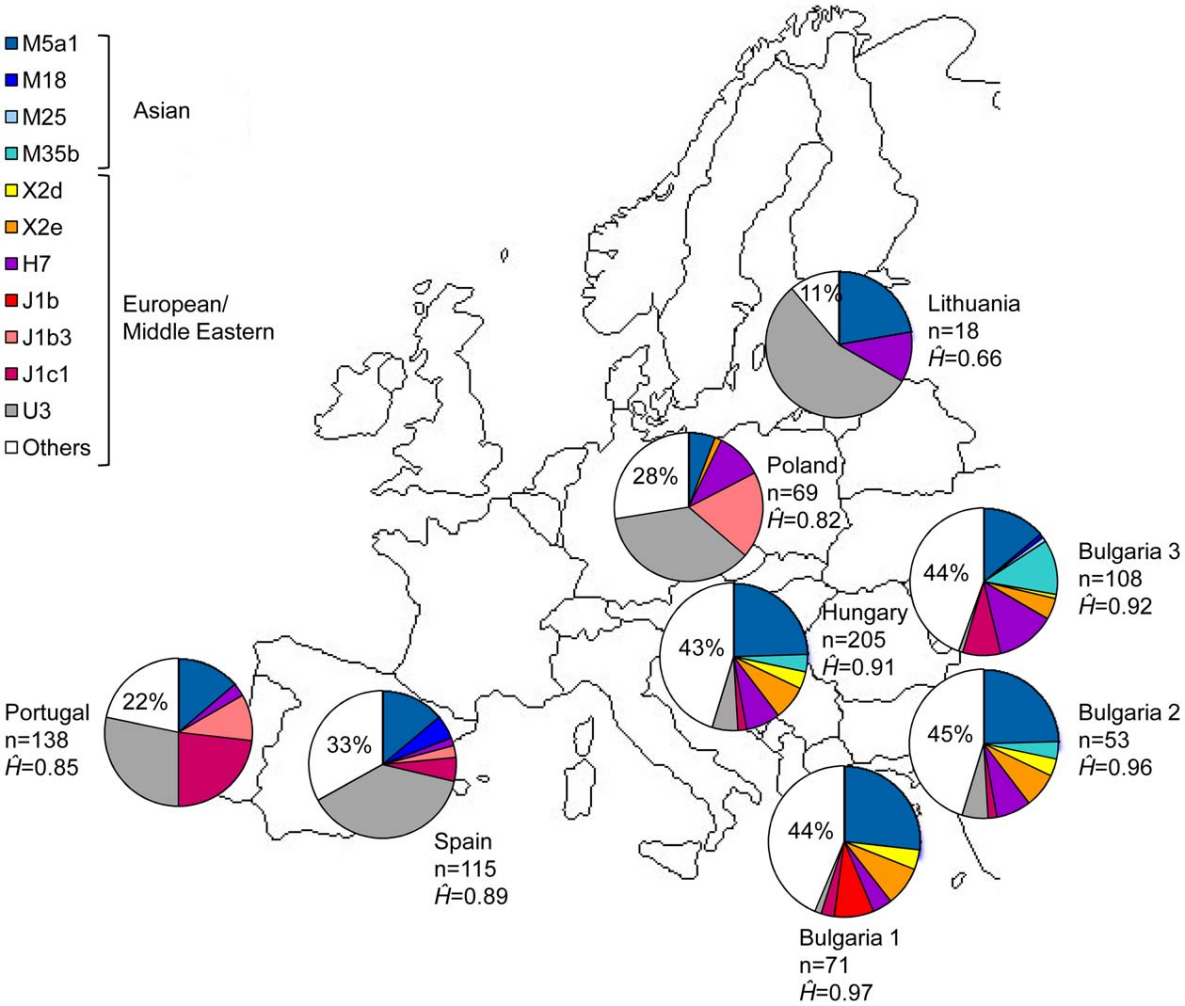
MtDNA haplogroups corresponding to founder lineages in the European Roma populations. Percentages of non-founder lineages are shown in white in the circles. Sample sizes (n) and sequence diversity (Ĥ) are shown for each Roma sample.

Summary statistics of the HVR-I for the Iberian Roma, other European Roma, and corresponding host populations from the literature are given in Table 1. Sequence diversity values for HVR-I were systematically lower in Roma groups compared to host populations (all distributions were not overlapping within two standard deviations, except in the Bulgarian Roma). A cline of diversity reduction was observed from Hungary and Balkans towards the Northern and Western edges of the Roma distribution in Europe (see also Figure 1). Mean pairwise differences were similar in Roma and non-Roma populations. However, WIMP values showed low levels of diversity within haplogroups in the Roma populations (2.2) in comparison to the host populations (2.9). These results suggest that the maternal gene pool in the Roma is composed of distantly related lineages with very low internal diversity.

**Table 1:** Sequence diversity indices for mtDNA lineages (positions 16,090–16,365) in the Roma populations and corresponding host populations included in the present study.

Population	N	K (%K)	S (%S)	*Ĥ*±sd	*π*±sd	*θ*_π_±sd	*θ*_K_ (95% CI)	*θ*_S_±sd	*D*	*F*_S_
Roma Portugal^a^	138	22 (15.94)	38 (13.77)	0.85±0.02	0.01±0.01	3.92±1.98	7.14 (4.38–11.35)	6.91±1.91	−1.30	−3.92
Roma Spaina,b,c	115	29 (38.26)	36 (13.77)	0.89±0.02	0.01±0.01	3.59±1.84	12.20 (7.80–18.70)	6.76±1.93	−1.43*	−12.83**
Roma Bulgaria1 c	71	35 (49.29)	36 (13.04)	0.97±0.01	0.02±0.01	4.97±2.45	26.70 (16.65–42.66)	7.45±2.25	−1.07	−21.24**
Roma Bulgaria2 c	53	23 (43.40)	29 (10.51)	0.96±0.01	0.02±0.01	4.57±2.53	14.90 (8.58–25.65)	6.39±2.07	−0.94	−8.69*
Roma Bulgaria3 c	108	31 (28.70)	35 (12.68)	0.92±0.01	0.02±0.01	4.40±2.43	14.20 (9.12–21.74)	6.66±1.92	−1.04	−12.44*
Roma Hungary^d^	205	43 (20.98)	50 (18.12)	0.91±0.01	0.02±0.01	6.05±2.89	16.30 (11.40–23.10)	8.48±2.15	−1.22	−17.97**
Roma Lithuania^c^	18	5 (27.77)	9 (3.26)	0.66±0.10	0.01±0.01	2.98±1.64	1.90 (0.70–5.10)	2.62±1.22	0.01	1.28
Roma Poland^e^	69	13 (18.84)	21 (7.61)	0.82±0.03	0.02±0.01	5.19±2.54	4.50 (2.40–8.10)	4.37±1.45	−0.03	0.08
Host Portugal^f^	118	81 (68.64)	60 (21.74)	0.97±0.01	0.01±0.01	4.21±2.10	112.73 (76.90–166.80)	11.23±2.97	−1.97**	−25.74**
Host Spain^g^	68	61 (89.71)	58 (21.01)	0.99±0.01	0.02±0.01	5.26±2.57	281.23 (142.90–591.60)	12.11±3.48	−1.89*	−25.46**
Host Bulgaria^h^	141	86 (60.99)	70 (25.36)	0.98±0.01	0.02±0.01	4.89±2.40	92.60 (65.90–130.80)	12.68±3.22	−2.12**	−25.74**
Host Hungary d	211	135 (69.59)	79 (28.62)	0.98±0.01	0.02±0.01	4.26±2.12	160.56 (120.84–214.07)	13.33±3.17	−2.07**	−25.45**
Host Lithuania^i^	162	96 (59.26)	72 (26.09)	0.98±0.01	0.02±0.01	5.63±2.72	98.20 (71.40–135.30)	12.72±3.16	−1.98*	−25.46**
Host Poland^j^	413	195 (47.21)	102 (36.96)	0.97±0.01	0.02±0.01	5.14±2.50	143.7 (117.20–176.00)	15.46±3.31	−2.13*	−25.13**

a Data from present study;

b Data from Fernandez et al. [20];

c Data from Gresham et al. [6];

d Data from Irwin et al. [7];

e Data from Malyarchuk et al. [9];

f Unpublished data;

g Data from Alvarez et al. [21];

h Data from Richards et al. [22];

i Data from Lappalainen et al. [23];

j Data from Grzybowski et al. [24].

*N*, sample size; *K*, number of different sequences; *S*, number of polymorphic sites; *Ĥ*, sequence diversity; *π*, nucleotide diversity; *θ*_π_ mean number of pairwise differences between sequences; *θ*_K_ mean number of pairwise differences based on *K*; *θ*_S_ mean number of pairwise differences based on *S*; *D*, Tajima's test of selective neutrality; *F*_S_, Fu's test of selective neutrality;

*P-value<0.05;

**P-value<0.001.

Signs of limited female effective population sizes for the Roma are evident from the values of the θ*_S_* and θ*_K_* estimators. Although both estimators were highly correlated in the Roma groups (r = 0.883, p-value<0.001), θ*_K_* is likely to provide a more reliable estimate of recent female effective-population size in the case of mtDNA control region [29]. θ*_K_* showed great disparity in Roma versus non-Roma populations, with the mean values being much lower in the Roma whose θ*_K_* distributions (95% CI) did not overlap with the non-Roma ones (Table 1). These results indicate that the female effective-population size of the Roma is substantially lower than in the corresponding European host populations. Among the Roma, both estimators θ*_K_* and θ*_S_* showed the lowest values in Lithuania and Poland, followed by the Roma from Iberia, while Hungary and Bulgaria presented the highest values. The neutrality tests Tajima's D and Fu's revealed significant evidences of population growth in all host populations, whereas in the Roma groups signatures of population expansion were only significant for the Spanish Roma (for both statistics) and for Hungarian and Bulgarian Roma (Fu's values). In general, these results show reduced diversity and lower effective population size in the Roma populations.

### Genetic relationships among Roma groups

The question of the migration routes of Roma within Europe remains very poorly addressed from the genetic point of view. Gresham et al. [6] applied AMOVA to investigate the apportionment of genetic diversity within Roma considering the following scenarios: i) Early settlement in Bulgaria (Bulgaria 1 in the present study); ii) North-West route (Lithuanian and Spanish Roma); iii) Wallachia/Moldavian Roma settled in Bulgaria in the early 17^th^ and 18^th^ centuries (Bulgaria 2 in the present study); iv) Wallachia/Moldavian Roma settled in Bulgaria in the late 19^th^ century (Bulgaria 3 in the present study). In this work we performed a serial AMOVA locating the newly available Roma populations in the context of the migration routes described by Gresham et al. [6] and further testing the possibility of having integrated new/independent routes.

First, in agreement with previous studies we found that the Roma are best classified according to the migration route rather than the country of residence (Table 2). Additionally, our results confirmed that the two Vlax groups from Moldo-Wallachia likely represent two independent migration routes as suggested in the original publication [6], whereas the Hungarian Roma are more closely related to the Bulgarian 2, which indicates that the same (or genetically closed) populations in Moldo-Wallachia were the source of the Hungarian and Bulgarian samples included in this study. The additional information provided by the coverage of the Iberian Roma in the present study (increased sample size from 14 to 253 individuals) confirmed that a North/West route had more statistical support than separated migration routes to Iberia and to Central/Northern Europe, a result that points towards a shared migration route by the Iberian, Polish and Lithuanian populations after the split from the Central European/Balkan Roma.

**Table 2:** AMOVA with mtDNA sequences from the Roma populations analyzed.

		Variance		
Grouping criteria	Groups	Among groups	Among populations within groups	Within populations
Total sample	19 populations in the original publications		7.02**	92.98**
Country	Portugal, Spain, Lithuania, Poland, Hungary, Bulgaria	3.77**	3.61**	92.62**
Historical migration^a^		4.99**	3.08**	91.92**
	Early settlement in Balkans: Bulgaria 1			
	Settlement in Bulgaria and Hungary from Wallachia/Moldavia 17^th^-18^th^ centuries: Bulgaria 2 and Hungary			
	Settlement in Bulgaria from Wallachia/Moldavia late 19^th^ century: Bulgaria 3			
	North/Western route: Lithuanian, Polish, Spanish, Portuguese Roma			

a The grouping showed explains the highest variance among groups (other results in Table S2).

*P-value<0.05;

**P-value<0.001.

According to the haplogroup composition, two different groups of lineages could be distinguished among the Roma. The European/Middle Eastern haplogroups accounted for 65% to 94% in different Roma groups, whereas the rest of the lineages belonged to haplogroup M. This last haplogroup is common in East Africa and Asia but it is rarely found in Europe [35]. Within haplogroup M, all lineages were of clear Asian origin except one East African M1a1 sequence found in two Portuguese Roma. The main Asian subhaplogroups found were M5a1, M18, M25 and M35b, which have been reported to have an Indian origin [36], [37], [38], [39], [40]. M5a1 was the most frequent Asian subhaplogroup found in all Roma populations (ranging from 6% to 29%). Most M5a1 sequences presented the HVR-I 16298C variant, which probably defines M5a1b since it was present in the two complete mitochondrial sequences studied by Malyarchuk et al [37]. M18 was mainly found in the Spanish Roma (5%) but one M18 individual was also reported in the Bulgarian Roma. Finally M35b, which was described in Bulgarian (Bulgaria 2, 4% and Bulgaria 3, 12%) and Hungarian Roma (5%), and M25 found in a Bulgarian individual.

Regarding the European/Middle Eastern haplogroups, we found seven lineages (see Figure 1) which are relatively common in Roma groups while they are atypical or low frequent in European populations according to the search of identical matches in the extended European host database of 5,096 sequences. These founder lineages represented less than half of the Roma individuals with non-M sequences (putative European origin), however they showed only 3% of the total matches in the database. Among them was U3, which despite being shared by all Roma studied it was found at particularly high frequencies in groups from Iberia, Lithuania and Poland. Such distribution pattern of U3 together with its extremely reduced internal diversity (Table S1) are again compatible with the hypothesis of a common out-of-the Balkans migration route of Iberian, Lithuanian and Poland Roma. Additionally, H7 was present in Roma from Iberia, Poland, Bulgaria and Lithuania; and J1b3 was found in all Roma except the Lithuanian. Moreover, two subtypes of X showed a more restricted distribution limited to Central Europe and Balkans: the subclade of X2e defined by 16241G and a subclade of X2d carrying the transversion 16189A. Finally, we were able to confirm the presence of haplogroup J1c1 in Roma from Iberia and Hungary and discard its presence in Poland. Since J1c1 ascription required information from the entire control region and this was not available for all Roma groups, we assigned HVR-I motives by identical matches of confirmed J1c1 sequences. Additionally, as we did with other lineages for which the HVR-I information was enough for the haplogroup classification, and aiming at assessing its frequency in the European host populations, we compared the complete control region of the Roma J1c1 individuals to the database of 7,330 total complete control sequences in EMPOP [41], and found no identical matches except with two sequences in the database which were from Hungarian Roma (Irwin, 2007).

Adding these seven lineages to the four M Asian subhaplogroups (M5a1, M18, M25 and M35b), it summed up to eleven lineages, which were absent or occurred at a very low frequency in the European host populations. Thus, these could represent founder lineages of European Roma, in the sense of being ancestral lineages widely shared by different European Roma groups. The diverse mtDNA resolution used in different studies did not permit to identify accurately other possible founder lineages. Nonetheless, M5a1, M18, M25, M35b, U3, H7, J1b, J1b3, J1c1, X2e and X2d represent the minimum component of ancestral lineages already present in the most ancient Roma groups settled in Europe. Probably most of these lineages were incorporated in the Roma before the arrival to the European continent.

### Genetic relationships between Roma and European host populations

The identification of founder lineages provided a rough estimate of the maximum percentage of admixture with European host populations that ranged from 11% in Lithuania to 45% in Bulgaria (see Figure 1). These values represented overestimates of the rate of admixture with host populations since the identification of other founder Roma lineages would require higher resolution. Indeed, founder Roma lineages that were also common in Europe would not be detected. Even so, it seems that most of the maternal lineages found in current Roma populations were already present in the most ancient Roma settled in Europe.

Next, we aimed to assign the origin of the putative European sequences found in the Roma within Europe by identical matches in the extended European host database (5,096 sequences). The objective of this analysis was to distinguish the relative contribution of local admixture of each of the Roma groups (Iberia, North, and Central/Balkan groups) with their specific host populations and in addition to distinguish between recent gene flow with current neighboring hosts from ancient gene flow related to the first entrance of Roma in Europe (admixture with Hungarian/Balkan hosts). However, the matching analysis showed that the putative European Roma sequences could be assigned to any region within Europe with similar probabilities (data not shown). This result highlighted the lack of phylogeographic resolution of European sequences at the sequence length considered in the current study.

**Figure 2:**
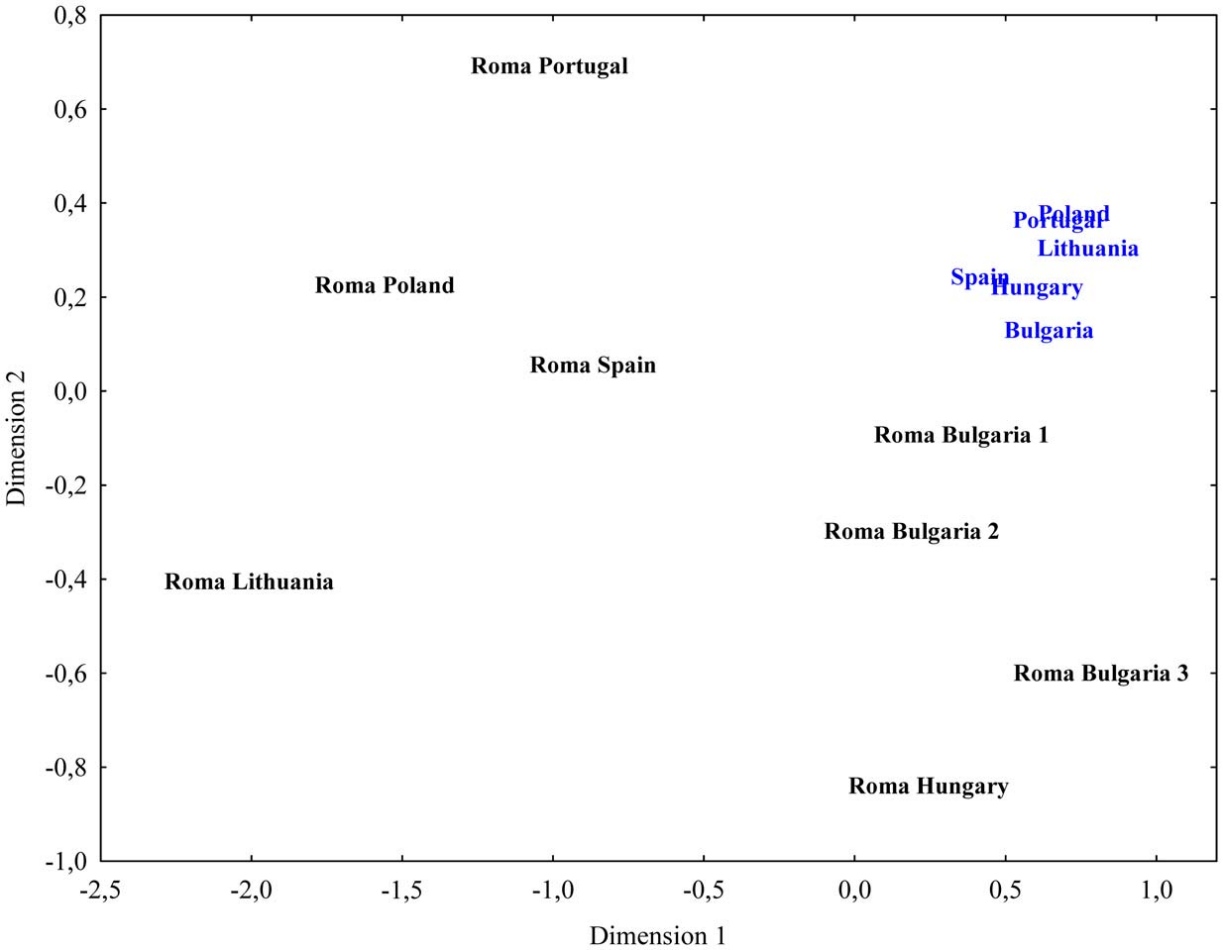
Non-metric Multidimensional Scaling plot (NMDS) of the pairwise differences between Roma and the corresponding host populations (stress value = 0.068). The labels “Roma Bulgaria 1”, “Roma Bulgaria 2” and “Roma Bulgaria 3” stand for Bulgarian Roma populations grouped according to history of migrations as in Gresham et al. [6].

In order to visualize the genetic affinities between different Roma and host populations, we built a non-metric Multidimensional Scaling (Figure 2, stress value = 0.068). The relative position of the populations in the two axes reflected higher internal heterogeneity between the Roma groups when compared to the host populations. The Roma appeared dispersed in the plot whereas the host populations were clustered together. In addition, the Roma populations did not show any preferential affinities with their respective host populations. This pattern is most probably explained by the intense drift undergone by the Roma groups, which might have shifted the allele frequencies of founder maternal lineages in the different Roma populations.

### Indian origins of the Roma

In an attempt to assign the origin of the Roma population to a specific geographical area in India, we searched for identical matches within the Roma sequences belonging to the Asian M sub-haplogroups (so then, the M1a1 lineage was not included). These lineages were compared to a dataset of 3,751 sequences from seven different regions and 22 states in India. Then, the weighted proportion of Roma sequences found in each geographical region or state were used to infer the probabilities of origin, which are listed in Table 3.

**Table 3:** Estimated probabilities for the subcontinental regions and states considered in the matching analysis and the corresponding standard deviations (SD).

Subcontinental region	State	n	Probability Region (SD)	Probability State (SD)
***North-western India***		418	0.721 (0.038)	
	Himachal Pradesh	37		0.017 (0.011)
	Kashmir	19		-
	Punjab	362		0.536 (0.042)
***Northern India***		314	0.022 (0.012)	
	Uttar Pradesh	232		0.014 (0.010)
	Madhya Pradesh	82		0.002 (0.003)
***Western India***		348	0.008 (0.007)	
	Guajarat	91		0.002 (0.004)
	Maharashtra	221		0.004 (0.005)
	Rajastan	36		-
***South-western India***		431	-	
	Karnataka	201		-
	Kerala	230		-
***South-eastern India***		1443	0.051 (0.019)	
	Tamil Nadu	427		0.003 (0.005)
	Andhra Pradesh	1016		0.033 (0.015)
***Eeastern India***		483	0.198 (0.034)	
	Bihar	45		0.058 (0.020)
	Orissa	153		0.299 (0.039)
	West Bengal	285		0.034 (0.015)
***North-eastern India***		314	-	
	Arunachal Pradesh	26		-
	Asma	58		-
	Manipur	9		-
	Mizoram	14		-
	Nagaland	43		-
	Tripura	134		-
	Bangladesh	30		-
	TOTAL	3751		

Those regions/states with no matches with Roma sequences (probability = 0) are shown with hyphens (-).

The subcontinental region showing higher probability of being the source of Roma sequences was North-western India (0.72), followed by Eastern India (0.20) while the rest of the subcontinental regions accounted only for 8% of the probability. When the analysis was performed at state level, results pointed at Punjab state (in North-Western India) as the most probable candidate to be the ancestral homeland of the Roma mtDNA types (probability = 0.54).

**Figure 3:**
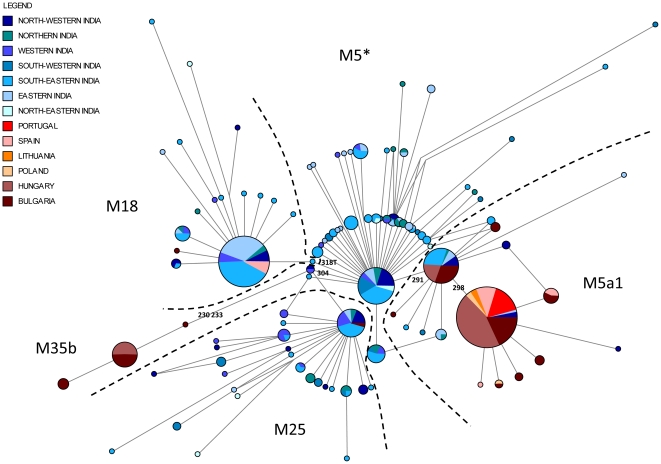
Median-joining network of the mtDNA sequences belonging to the M5*, M51a, M25, M35 and M18 haplogroups in the Roma and Indian populations (numbers represent mutation defining these haplogroups).

Finally, we built a median-joining network to compare M5a1, M18, M25 and M35b lineages found in the Roma to the Indian ones. In all cases, the Roma from Hungary and Bulgaria showed higher sequence diversity than the populations located in the edges of the Roma migration routes (see Figure 3). For all subhaplogroups except M35b, Roma sequences represented a subset of the total diversity found in India, showing clear signs of founder effects within the Roma. This was especially noticeable in subhaplogroup M5a1, which was found at high frequency in all Roma samples but being much less common in India. The lack of representation of some of the Roma M5a1 and M35b sequences in the Indian dataset might reflect the current under-sampling state of mtDNA diversity within the sub-continent, which may be particularly critical for certain Indian tribes and castes. Despite that, it seems likely that the diversification of M5a1 and M35b has been highly private to the Roma populations. As for M5a1, our results suggested that the original gene pool of the proto-Roma was greatly enriched in these lineages, some of which – those harbouring 16298C- might have increased in frequency before its diversification. Admitting that the diversification of subhaplogroup M5a1 mainly occurred after the Roma had left India, the time estimate of this lineage may provide a rough upper limit of the timing of the Roma exodus. Under this hypothetical scenario and assuming 16129A-16223T-16291T-16298C to be the putative ancestral haplotype, the TMRCA of the Romani M5a1 lineages was estimated at 2,158±1,178 years in agreement with previous historical records that locate the Roma in Europe at least 1,000 years ago [3].

## Discussion

The pattern of mtDNA diversity in the Roma from Europe retains remarkable signs of their recent demographic past. By the fourteenth century, many Roma groups are recorded to be established in the Balkan Peninsula. Departing from this region, a chain of group fragmentation and migration events would have lead to their spread throughout Europe, in such a large-scale and fast movement that only one century elapsed until their presence was documented even in the most peripheral regions of Europe, from the North-east to the South-west corners of the continent. It is acknowledged that during this itinerant period, Roma usually travelled in small groups before the arrival and settlement in new places, from where often new waves of branching and migration across the region initiated [2], [42].

In agreement with these accounts, our results show that the maternal effective population sizes in the Roma are strikingly low in comparison to the host populations. Whereas host European populations and Indians [32] show strong molecular signatures of population expansion, Roma groups remained constant. Additionally, populations which have experienced one or several founder events are expected to show lower θ*_K_* values than those from the source populations. Since Indian populations tend to exhibit much higher values than the European hosts here considered [32], the demographic parameters found in Roma testify strong signals of founder effects compared to both putative parental populations. Of note that among the European Roma diversity decreases from Eastern (represented by the Bulgarian and Hungarian Roma) towards Western and Northern groups, fitting an expectable accumulation of drift effects during successive population splitting and migrations along the dispersion of Roma within Europe.

Despite the persistence of founder Romani maternal lineages in different Roma groups, bottleneck events profoundly drifted the frequency of haplogroups contained in the ancestral pool, contributing to generate strong differentiation between groups.

Our results further suggest that the Iberian, Polish, and Lithuanian Roma were derived from the same migration wave, which, probably due to the low effective population size of fragmented groups, resulted in strong differentiation from the Central/Balkan Roma from which it was originated. This differentiation process implied the loss of lineages in parallel with a random dramatic increase of other ones. The random accumulation of founder effects does not permit the accurate identification of all possible founder lineages in the European Roma, since many of them may be present at low frequencies in the Balkan Roma but absent due to loss in Roma from other regions in Europe.

Even so, the conservative identification of the founder lineages M5a1, M18, M25, M35b, U3, H7, J1b, J1b3, J1c1, X2e and X2d allowed us to obtain maximum admixture proportions with host populations. Overall, the incorporation of female lineages from non-Roma appears to have been low since most of the sequences present in current Roma are rare in the European host populations, suggesting that the majority of lineages were already present in Roma before their arrival in Europe. The phylogeography of the Roma founder lineages demonstrates a broad West Eurasian origin (except those belonging to macrohaplogroup M) not confined to Western Europe. In fact, haplogroups such HV, pre-HV, J-T, U-K, I, W and X are present in highest frequencies in the Anatolian/Caucasus and Iranian regions [38] being moreover still present at relatively high frequencies in the Indus Valley and Central Asia [38], [43]. Given this distribution, higher phylogeographic resolution is needed to distinguish among lineages from such a broad geographical area.

The upper limits of admixture rates in the maternal genetic pool of the Roma range from low (11%) to moderate (50%). Unfortunately, similar studies on paternal lineages of European Roma populations are confined to the Iberian Peninsula [11]. Our estimates of maternal admixture in Iberian Roma (30%) are slightly lower than estimates for the Y-chromosome (47%) reported by Gusmão et al. Anthropological records show that marriages with non-Roma are usually avoided in the Roma communities [1], although non-Roma females are more frequently accepted in the Roma groups than non-Roma males [44], [45]. Unexpectedly, we detect less percentage of admixture rates in the maternal pool than that reported on paternal lineages. However, the high values for both estimates show that the amount of admixture observed contradict the stereotype of Roma constituting closed endogamous groups. Our results may indicate that social rules practiced by the Roma may have been varying in time and space according to different social constrains. Nevertheless, the proportions of admixture in the maternal and paternal genetic pools have to be considered rough approximations since they depend on the phylogeographic resolution on the mtDNA sequences and Y-chromosome haplotypes. Further studies providing better phylogeographic resolution and better coverage of Indian and European populations may give more accurate estimates of admixture rates. This would lead to confirm if asymmetry exists between maternal and paternal lineages and whether different European Roma groups show similar patterns.

In contrast, the more restricted phylogeography of haplogroup M points to the Indian subcontinent as the origin of a substantial fraction of Roma maternal lineages. A match analysis with the Roma M-founder lineages using a database of more than 3,700 Indian sequences, allowed us to identify North-Western India, and specifically the Punjab region, as the putative homeland of the Roma Diaspora. This finding is in accordance with previous linguistic and cultural evidences [4], as well as with the recent genetic hint provided by the identification of a private mutation in the Roma shared by a Jatti family in the Punjab province of Pakistan [14]. To our knowledge, this is the first comprehensive study comparing different Indian subcontinental areas in order to assess the origin of the Roma. Better coverage of India and surrounding areas in future studies will allow to determine the contribution of different tribes or castes from the Punjab area to the ancient Roma population who left India.

In summary, our findings confirm the high genetic heterogeneity of the Roma groups which has been shaped by several founder events combined with low effective population sizes, creating a pattern that mimics the migration routes the Roma followed within Europe. We show that most maternal Roma lineages are of non-European origin, pointing to a limited admixture with surrounding populations. Finally, the phylogeographic information provided by the Indian female lineages found in the Roma led us to trace back the ancient homeland of the European Roma to the Punjab state, in North-western India, confirming previous linguistic and anthropological accounts.

## Supporting Information

Table S1 **Polymorphic positions for mtDNA complete control region present in the Iberian Roma.**
(DOC)

Table S2 **Serial AMOVA locating the populations with unknown historical migrations in the migration routes previously described by Gresham et al.** [**1**] **.** *P-value<0.05; **P-value<0.001.
(DOC)
