# Supplementary material for: Reconstructing the Indian Origin and Dispersal of the European Roma: A Maternal Genetic Perspective
Source: PLoS One. 2011 Jan 10;6(1):e15988. doi: 10.1371/journal.pone.0015988 (PMC3018485; doi:10.1371/journal.pone.0015988)
Supplement: Table S1 — Polymorphic positions for mtDNA complete control region present in the Iberian Roma. (DOC) [file pone.0015988.s001.doc]

Table S1. Polymorphic positions for mtDNA complete control region present in the Iberian Roma.

| **Sample ID** | **Polymorphic sites (16024-576)** | **Haplogroup** | **N RoPor** | **N RoBarc** | **SNP*** |
| --- | --- | --- | --- | --- | --- |
| **Hap1** | 16218 16278 93 263 315.1C | H | 0 | 1 | 750; 4769 |
| **Hap2** | 16218 16328 16362 249DEL 263 292 315.1C | H20a | 0 | 1 | 750; 4769 |
| **Hap3** | 16223 16519 263 315.1C | H | 1 | 0 | 750; 1438; 4769 |
| **Hap4** | 16223 16519 263 315.1C | H | 0 | 1 | 750; 4769 |
| **Hap5** | 16092 16169 16293 16311 263 315.1C | H11 | 1 | 0 | 750; 1438; 4769; 6776; 13759 |
| **Hap6** | 16519 263 315.1C | H | 1 | 0 | 750; 1438; 4769 |
| **Hap7** | 16519 146 263 315.1C 574.1C 574.2C 574.3C 574.4C 574.5C 574.6C | H3c | 1 | 0 | 750; 1438; 4769; 6776; 12957 |
| **Hap8** | 16304 16519 93 263 315.1C 456 513 | H5a | 0 | 1 | 750; 4336 |
| **Hap9** | 16304 16519 93 263 315.1C 456 513 | H5a | 0 | 2 | 750; 4336; 4769 |
| **Hap10** | 16129 16362 16482 239 263 315.1C | H6 | 1 | 0 | - |
| **Hap11** | 16129 16362 16482 239 263 315.1C | H6a1 | 2 | 0 | 1438; 3915; 4727; 4769 |
| **Hap12** | 16261 16519 263 309.1C 309.2C 315.1C | H7a1 | 0 | 1 | 750; 4769; 4793 |
| **Hap13** | 16261 16304 16519 93 263 315.1C 523DEL 524DEL | H7a1 | 1 | 0 | 1438; 4769; 4793 |
| **Hap14** | 16261 16304 16519 93 263 315.1C 523DEL 524DEL | H7a1 | 3 | 0 | 1438; 4793 |
| **Hap15** | 16261 16304 16519 93 263 315.1C 523DEL 524DEL | H7a1 | 0 | 1 | *n.d.* |
| **Hap16** | 16298 16390 16519 263 315.1C | HV0 | 0 | 1 | *n.d.* |
| **Hap17** | 16298 16390 16519 263 309.1C 315.1C | HV0 | 0 | 1 | *n.d.* |
| **Hap18** | 16298 72 195 263 309.1C 315.1C | HV0 | 0 | 1 | *n.d.* |
| **Hap19** | 16298 16519 72 263 315.1C | HV0 | 5 | 0 | *n.d.* |
| **Hap20** | 16153 16298 72 195 198 263 315.1C | HV0b | 1 | 0 | *n.d.* |
| **Hap21** | 16069 16126 16278 16366 16519 73 185 188 228 263 295 315.1C 462 489 523DEL 524DEL | J1c2 | 0 | 3 | *n.d.* |
| **Hap22** | 16069 16126 73 185 188 228 263 295 315.1C 462 489 524.1A 524.2C | J1c | 1 | 0 | *n.d.* |
| **Hap23** | 16069 16126 73 185 228 263 295 315.1C 462 482 489 508 | J1c1 | 31 | 0 | *n.d.* |
| **Hap24** | 16069 16126 73 185 228 263 315.1C 462 482 489 508 | J1c1 | 0 | 5 | *n.d.* |
| **Hap25** | 16069 16126 16145 16222 16235 16261 16271 73 263 295 309.1C 315.1C 462 489 | J1b3 | 0 | 1 | *n.d.* |
| **Hap26** | 16069 16126 16145 16222 16235 16261 16271 16519 73 146 263 295 315.1C 462 489 | J1b3 | 1 | 0 | *n.d.* |
| **Hap27** | 16069 16126 16145 16222 16235 16261 16271 16519 73 263 295 309.1C 315.1C 462 489 | J1b3 | 7 | 0 | *n.d.* |
| **Hap28** | 16069 16126 16145 16222 16235 16261 16271 16519 73 263 295 315.1C 462 489 | J1b3 | 6 | 0 | *n.d.* |
| **Hap29** | 16069 16126 16193 73 150 152 263 295 315.1C 489 508 523DEL 524DEL | J2b | 0 | 3 | *n.d.* |
| **Hap30** | 16069 16126 16193 16278 16519 73 150 152 263 295 309.1C 315.1C 489 | J2b1a | 1 | 0 | *n.d.* |
| **Hap31** | 16069 16126 16193 16278 73 150 152 263 295 309.1C 309.2C 315.1C 489 499 | J2b1a | 1 | 0 | *n.d.* |
| **Hap32** | 16224 16311 16519 73 263 315.1C 497 | K1a | 1 | 0 | *n.d.* |
| **Hap33** | 16093 16224 16311 16319 16463 16519 73 152 263 315.1C 524.1A 524.2C | K1b1a1 | 0 | 3 | *n.d.* |
| **Hap34** | 16223 16318T 16519 73 93 246 309.1C 263G 315.1C 489 | M18 | 0 | 5 | *n.d.* |
| **Hap35** | 16129 16189 16223 16249 16311 16359 16519 73 195 263 315.1C 489 | M1a1 | 2 | 0 | *n.d.* |
| **Hap36** | 16129 16148 16223 16291 16298 16519 73 263 310 315.1C 489 524.1A 524.2C | M5a1 | 0 | 3 | *n.d.* |
| **Hap37** | 16129 16223 16291 16298 16519 73 263 309.1C 315.1C 489 | M5a1 | 2 | 0 | *n.d.* |
| **Hap38** | 16129 16223 16291 16298 16519 73 263 309.1C 315.1C 489 524.1A 324.1C | M5a1 | 17 | 1 | *n.d.* |
| **Hap39** | 16129 16223 16291 16298 16519 73 263 309.1C 309.2C 315.1C 489 524.1A 524.2C | M5a1 | 0 | 3 | *n.d.* |
| **Hap40** | 16129 16223 16291 16298 16519 73 263 309.1C 315.1C 489 | M5a1 | 0 | 1 | *n.d.* |
| **Hap41** | 16129 16223 16291 16298 16519 73 263 309.1C 309.2C 315.1C 489 | M5a1 | 0 | 1 | *n.d.* |
| **Hap42** | 16126 16153 16294 16296 16519 73 150 263 315.1C | T2e | 0 | 1 | *n.d.* |
| **Hap43** | 16126 16343 73 150 263 315.1C | U3 | 1 | 0 | *n.d.* |
| **Hap44** | 16260 16343 73 150 263 315.1C | U3 | 0 | 6 | *n.d.* |
| **Hap45** | 16343 73 150 189 263 315.1C | U3 | 1 | 0 | *n.d.* |
| **Hap46** | 16343 73 150 204 263 315.1C | U3 | 1 | 0 | *n.d.* |
| **Hap47** | 16189 16343 73 150 263 315.1C | U3 | 1 | 0 | *n.d.* |
| **Hap48** | 16343 73 150 263 309.1C 315.1C | U3 | 5 | 0 | *n.d.* |
| **Hap49** | 16343 73 150 263 315.1C | U3 | 30 | 21 | *n.d.* |
| **Hap50** | 16256 16343 16390 16519 73G 150 152 263 309.1C 315.1C 524.1A 524.2C | U3a | 0 | 2 | *n.d.* |
| **Hap51** | 16179 16356 16519 73 195 263 309.1C 315.1C 499 | U4c1 | 1 | 0 | *n.d.* |
| **Hap52** | 16167 16192 16270 16311 16356 73 150 263 309.1C 315.1C | U5b | 1 | 0 | *n.d.* |
| **Hap53** | 16183C 16187 16189 16192 16270 73 150 195 263 315.1C | U5b | 0 | 2 | *n.d.* |
| **Hap54** | 16192 16311 73 150 263 309.1C 315.1C | U5b | 0 | 1 | *n.d.* |
| **Hap55** | 16270 16292 16362 73 150 263 309.1C 315.1C | U5b | 1 | 0 | *n.d.* |
| **Hap56** | 16129 16172 16219 73 263 315.1C | U6 | 0 | 3 | *n.d.* |
| **Hap57** | 16172 16183C 16189 16219 16239 16278 73 263 309.1C 315.1C 523DEL 524DEL | U6a1 | 3 | 0 | *n.d.* |
| **Hap58** | 16172 16174 16188 16219 16311 73 263 315.1C | U6 | 1 | 0 | *n.d.* |
| **Hap59** | 16146 16342 73 263 282 309.1C 315.1C | U8a1 | 5 | 0 | *n.d.* |
|  |  | TOTAL | 138 | 76 |  |

RoPor, Roma from Portugal; RoBarc, Roma from Barcelona, Spain.

* SNPs derived for the mutations defining H subhaplogroup as in Álvarez-Iglesias et al. [1]; *n.d.*, not determined;

References

1. Alvarez-Iglesias V, Mosquera-Miguel A, Cerezo M, Quintans B, Zarrabeitia MT, et al. (2009) New population and phylogenetic features of the internal variation within mitochondrial DNA macro-haplogroup R0. PLoS One 4: e5112.
