# Supplementary material for: Reconstructing the Indian Origin and Dispersal of the European Roma: A Maternal Genetic Perspective
Source: PLoS One. 2011 Jan 10;6(1):e15988. doi: 10.1371/journal.pone.0015988 (PMC3018485; doi:10.1371/journal.pone.0015988)
Supplement: Table S2 — Serial AMOVA locating the populations with unknown historical migrations in the migration routes previously described by Gresham et al. [1] . *P-value<0.05; **P-value<0.001. (DOC) [file pone.0015988.s002.doc]

Table S2. Serial AMOVA locating the populations with unknown historical migrations in the migration routes previously described by Gresham et al. [1]. **P*-value < 0.05*; **P*-value < 0.001.

|  |  | **Variance** | | |
| --- | --- | --- | --- | --- |
| **Grouping criteria** | **Groups** | **Among**  **groups** | **Among populations within groups** | **Within**  **populations** |
| **History_migrations** |  |  |  |  |
| 1 |  | 4.81** | 2.92** | 92.27** |
|  | Early settelment in Bulgaria and Hungary |  |  |  |
|  | Wallachia/Moldavia 17-18th centuries |  |  |  |
|  | Wallachia/Moldavia late 19th century |  |  |  |
|  | Iberia(Portugal and Spain) |  |  |  |
|  | North (PolandandLithuania) |  |  |  |
| 2 |  | 4.96** | 2.72** | 92.32** |
|  | Early settelment in Bulgaria |  |  |  |
|  | Wallachia/Moldavia 17-18th centuries and Hungary |  |  |  |
|  | Wallachia/Moldavia late 19th century |  |  |  |
|  | Iberia(Portugal and Spain) |  |  |  |
|  | North (Poland and Lithuania) |  |  |  |
| 3 |  | 3.67** | 3.99** | 92.34** |
|  | Early settelment in Bulgaria |  |  |  |
|  | Wallachia/Moldavia 17-18th centuries |  |  |  |
|  | Wallachia/Moldavia late 19th century and Hungary |  |  |  |
|  | Iberia(Portugal and Spain) |  |  |  |
|  | North (Poland andLithuania) |  |  |  |
| 4 |  | 4.57** | 3.22** | 92.22** |
|  | Early settelment in Bulgaria and Hungary |  |  |  |
|  | Wallachia/Moldavia |  |  |  |
|  | Iberia(Portugal and Spain) |  |  |  |
|  | North (Poland andLithuania) |  |  |  |
| 5 |  | 4.23** | 3.77** | 92** |
|  | Early settelment in Bulgaria |  |  |  |
|  | Wallachia/Moldavia and Hungary |  |  |  |
|  | Iberia(Portugal and Spain) |  |  |  |
|  | North (Poland andLithuania) |  |  |  |
| 6 |  |  |  |  |
|  | Early settelment in Bulgaria and Hungary | 4.86** | 3.25** | 91.89** |
|  | Wallachia/Moldavia 17-18th centuries |  |  |  |
|  | Wallachia/Moldavia late 19th century |  |  |  |
|  | North/west route (Portugal,Spain,Lithuania,Poland) |  |  |  |
| 7 |  | 4.68** | 3.49** | 91.83** |
|  | Early settelment in Bulgaria and Hungary |  |  |  |
|  | Wallachia/Moldavia |  |  |  |
|  | North/west route (Portugal,Spain,Lithuania,Poland) |  |  |  |
| 8 |  | 4.46** | 3.95** | 91.6** |
|  | Early settelment in Bulgaria |  |  |  |
|  | Wallachia/Moldaviaand Hungary |  |  |  |
|  | North/west route(Portugal,Spain,Lithuania,Poland) |  |  |  |
| 9 |  | 3.83** | 4.16** | 92.02** |
|  | Early settelment in Bulgaria |  |  |  |
|  | Wallachia/Moldavia 17-18th centuries |  |  |  |
|  | Wallachia/Moldavia late 19th century and Hungary |  |  |  |
|  | North/west route (Portugal,Spain,Lithuania,Poland) |  |  |  |
| 10 |  | 1.91* | 5.91** | 92.18** |
|  | Early settelment in Bulgaria |  |  |  |
|  | Wallachia/Moldavia 17-18th centuries |  |  |  |
|  | Wallachia/Moldavia late 19th century |  |  |  |
|  | North/west route (Portugal,Spain,Lithuania,Poland) and Hungary |  |  |  |
| 11 |  | 4.61** | 3.21** | 92.18** |
|  | Early settelment in Bulgaria |  |  |  |
|  | Wallachia/Moldavia 17-18th centuries |  |  |  |
|  | Wallachia/Moldavia late 19th century |  |  |  |
|  | North/west route (Portugal,Spain,Lithuania,Poland) |  |  |  |
|  | Hungary |  |  |  |
| 12 |  | 2.74** | 4.85** | 92.41** |
|  | Early settelment in Bulgaria |  |  |  |
|  | Wallachia/Moldavia 17-18th centuries and Hungary and Poland |  |  |  |
|  | Wallachia/Moldavia late 19th century |  |  |  |
|  | North/west route (Portugal,Spain,Lithuania,Poland) |  |  |  |
| 13 |  | 4.87** | 2.86** | 92.27** |
|  | Early settelment in Bulgaria |  |  |  |
|  | Wallachia/Moldavia 17-18th centuries and Hungary |  |  |  |
|  | Wallachia/Moldavia late 19th century |  |  |  |
|  | North/west route (Iberia and Lithuania) |  |  |  |
|  | Poland |  |  |  |
| 14 |  | 3.51** | 4.08** | 92.41** |
|  | Early settelment in BulgariaandPoland |  |  |  |
|  | Wallachia/Moldavia 17-18th centuries and Hungary |  |  |  |
|  | Wallachia/Moldavia late 19th century |  |  |  |
|  | North/west route (Iberia and Lithuania) |  |  |  |
| 15 |  | 2.84** | 4.67** | 92.49** |
|  | Early settelment in Bulgaria |  |  |  |
|  | Wallachia/Moldavia 17-18th centuries and Hungary |  |  |  |
|  | Wallachia/Moldavia late 19th centuryandPoland |  |  |  |
|  | North/west route (Iberia and Lithuania) |  |  |  |
| 16 |  | 3.56** | 3.98** | 92.46** |
|  | Early settelment in Bulgaria and Poland |  |  |  |
|  | Wallachia/Moldavia 17-18th centuries and Hungary |  |  |  |
|  | Wallachia/Moldavia late 19th century |  |  |  |
|  | Iberia (Portugal and Spain) |  |  |  |
|  | Lithuania |  |  |  |

**References**

1. Gresham D, Morar B, Underhill PA, Passarino G, Lin AA, et al. (2001) Origins and divergence of the Roma (gypsies). Am J Hum Genet 69: 1314-1331.
